# Prevalence and Risk Factors of High-Risk Population for Stroke: A Population-Based Cross-Sectional Survey in Southwestern China

**DOI:** 10.3389/fneur.2022.693894

**Published:** 2022-03-02

**Authors:** Xingyang Yi, Hong Chen, Yanfen Wang, Ming Yu, Hua Luo, Chun Wang, Wei Wei, Xiaorong Chen, Shaozhi Bao

**Affiliations:** ^1^Department of Neurology, The People's Hospital of Deyang City, Deyang, China; ^2^Department of Neurology, The Suining Central Hospital, Suining, China; ^3^Department of Neurology, The Affiliated Hospital of Southwest Medical University, Luzhou, China; ^4^Department of Neurology, The Third Affiliated Hospital of Wenzhou Medical University, Wenzhou, China

**Keywords:** high risk stroke population, stroke, epidemiology, risk factors, health care

## Abstract

With the aging of the population and the change of lifestyle in China, the prevalence and risk factors of the high-risk population for stroke may change. However, few studies performed community-based high-risk stroke population surveys in China. Hence, we performed this multicenter, cross-sectional, and community-based survey in Sichuan of southwestern China, according to the China National Stroke Screening Survey (CNSSS) program in 2015. The residents aged ≥ 40 years volunteered to participate in the face-to-face survey in 8 communities in Sichuan. The 8 communities were selected using the cluster randomization method. Subjects with at least three of the eight stroke-related risk factors were classified as a high-risk population for stroke. Otherwise were classified as a low-risk population for stroke. Furthermore, 1,011 individuals were randomly selected among the low-risk population for stroke as controls. Among 16,892 participants, 2,369 (14.0%) were high-risk population for stroke. After full adjustments, all eight risk factors for stroke were significantly associated with the high-risk population for stroke (*P* < 0.001). The largest contributor was hypertension [population-attributable risk (PAR) 28.4%], followed by physical inactivity (PAR 14.2%), dyslipidemia (PAR 11.2%), overweight/obesity (PAR 9.4%), diabetes (PAR 7.2%), family history (PAR 6.8%), smoking (PAR 5.9%), and atrial fibrillation (PAR 3.5%). Carotid atherosclerosis was more serious in the high-risk population for stroke than in controls (*P* < 0.05). The prevalence of the high-risk population for stroke was high in southwestern China. Hypertension, physical inactivity, and dyslipidemia were stronger contributors for the high-risk population for stroke. Individual-level and population-level interventions for these leading risk factors are very important for the primary prevention of stroke.

## Introduction

Stroke is the leading cause of death and disability in China (1–3). In the past four decades, the incidence of stroke has decreased because of effective strategies for preventing stroke risk factors and good healthcare services in developed countries. However, the incidence of stroke has increased because of insufficiently primary prevention of stroke in developing countries (3). According to a report from the world health organization, the incidence of stroke in China is still increasing at an annual rate of 8.7% (4).

China has experienced rapid sociodemographic changes and health transitions in the past three decades; these may result in changes in the prevalence of traditional risk factors for stroke (2, 5). For example, there was a large increase in the prevalence of hypertension, smoking, overweight, dyslipidemia, diabetes mellitus, physical inactivity, diets low in fruit and vegetables, and high sodium intake (6–11); these are the most common and modifiable risk factors for stroke and all of these may affect stroke burden in China (12). Hypertension is the most important risk factor for stroke, and it was substantially increased in the past four decades in China (13). However, the standard-reaching rate of hypertension treatment is under 20% in China, this is significantly lower than in the high-income countries (14, 15). Similarly, diabetes and dyslipidemia are relatively common and poorly controlled in China (16). Large prospective studies revealed that lifestyle (physical inactivity and smoking) and dietary habits (such as high salt intake) were associated with the risk of stroke (6, 12).

The incidence of stroke is higher in individuals with multiple risk factors for stroke (high-risk population for stroke) than those individuals with health or low-risk population for stroke (1–3, 6, 12). The high incidence of stroke indicates that primary prevention strategies are insufficiently effective in China. Effective control of risk factors for stroke requires more effective public education and greater responsibilities of individuals. In addition, it is very important to screen out individuals with a high risk for stroke. Based on current progress and ongoing challenges, the Chinese government launched the China National Stroke Screening Survey (CNSSS) and intervention for high-risk population programs among residents of all the 31 provinces in China (12, 17).

Sichuan province is located in southwestern China, is an intermediate economic development area. The incidence of stroke was higher in Sichuan than in other regions, according to the CNSSS and previous studies (6, 12, 17, 18). However, epidemiological data for stroke in Sichuan were collected in the 1990s (19, 20), rare studies have revisited this important public health issue. In the past several decades, the Chinese lifestyle has greatly changed, and the aging population has increased, which has led to changes in the prevalence of stroke-related risk factors. Hence, we performed this community-based high-risk population for stroke survey in 8 communities in Sichuan according to the CNSSS program in 2015. This survey aimed to investigate the prevalence of high-risk population for stroke and the contribution of stroke-related risk factors for the high-risk stroke population and promote primary prevention of stroke.

## Materials and Methods

### Study Design and Participants

This population-based cross-sectional survey was part of the CNSSS program, which was supervised by the Chinese National Center for Stroke Care Quality Control and Management. The survey protocol was reviewed and approved by the Ethics Committee of the participating hospitals (IRB number: 2015-024) (the People's Hospital of Deyang City, the Affiliated Hospital of Southwest Medical University, and Suining Central Hospital) and informed consent was obtained from all the participants during recruitment.

The survey was conducted in the eight communities in Sichuan from May 2015 to September 2015. A cluster survey method was used and the eight communities were selected using the cluster randomization method. Details on the organization and implementation of the CNSSS can be found on the official website (21). Briefly, we screened residents for age ≥ 40 years in each community (22). All participants were people who had lived in the county (or district) for at least 6 months and were screened using a structured face-to-face questionnaire by interviewers. The questionnaire included demographic characteristics (e.g., age, gender, education level, and employment), stroke-related behavioral factors (e.g., smoking, exercise habits, and diet), personal and family medical history of stroke and chronic diseases [i.e., hypertension, diabetes mellitus, dyslipidemia and atrial fibrillation (AF)], and physical examination (e.g., height, weight, and resting blood pressure). More detailed information regarding the lifestyle, related diseases, and laboratory examinations [such as fasting blood glucose (FBG), lipid, ECG, and carotid ultrasonography] was obtained from the individuals who were identified to be at a high-risk population for stroke.

### Evaluation of Risk Factors

Because the main aims of the survey were to investigate the prevalence of high-risk populations for stroke and the contribution of stroke-related risk factors for the high-risk population. Therefore, by definition, the patients with a history of stroke or transient ischemic attack were not included in this analysis.

The eight risk factors for stroke were evaluated, namely, overweight/obesity, smoking, physical inactivity, family history of stroke, hypertension, diabetes, dyslipidemia, and AF. Hypertension was defined as a self-reported history or the use of antihypertensive drugs or the average of two resting systolic blood pressure readings of ≥ 140 mm Hg and/or diastolic blood pressure ≥ 90 mm Hg (23). Diabetes mellitus was defined as the use of insulin and/or oral hypoglycemic medications or a self-reported history of diabetes or FBG ≥ 7.0 mmol/l (24). Dyslipidemia was defined as using a lipid-lowering medication or having one or more of the following in the field survey: triglycerides (TGs) ≥ 1.70 mmol/l, cholesterol (TC) ≥ 5.18 mmol/l and low-density lipoprotein cholesterol (LDL-C) ≥ 3.37 mmol/l (25). AF was defined as reported by the respondent or diagnosed by ECG. Current smoking (≥1 cigarette per day) was defined by subjects' self-report. Body mass index (BMI) was calculated as weight (kg) divided by height squared (m^2^) and overweight/obesity was defined as BMI ≥ 26 kg/m^2^ (26). Physical inactivity was defined as physical exercise <3 times a week for <30 min each time (27). A family history of stroke was restricted to immediate family members.

Subjects with at least three of the aforementioned eight risk factors were classified as a high-risk population for stroke. Subjects with fewer than three of these risk factors were classified as low-risk stroke populations (21, 28). The risk assessment scales were designed by the CNSSS and have been proved to have good reliability and validity compared with the modified scale of the Framingham Stroke Profile, and can be used as an evaluation tool for stroke risk assessment (29).

### Data Cleaning Procedures, Quality Control, and Selection of Low-Risk Population for Stroke

**Figure 1:**
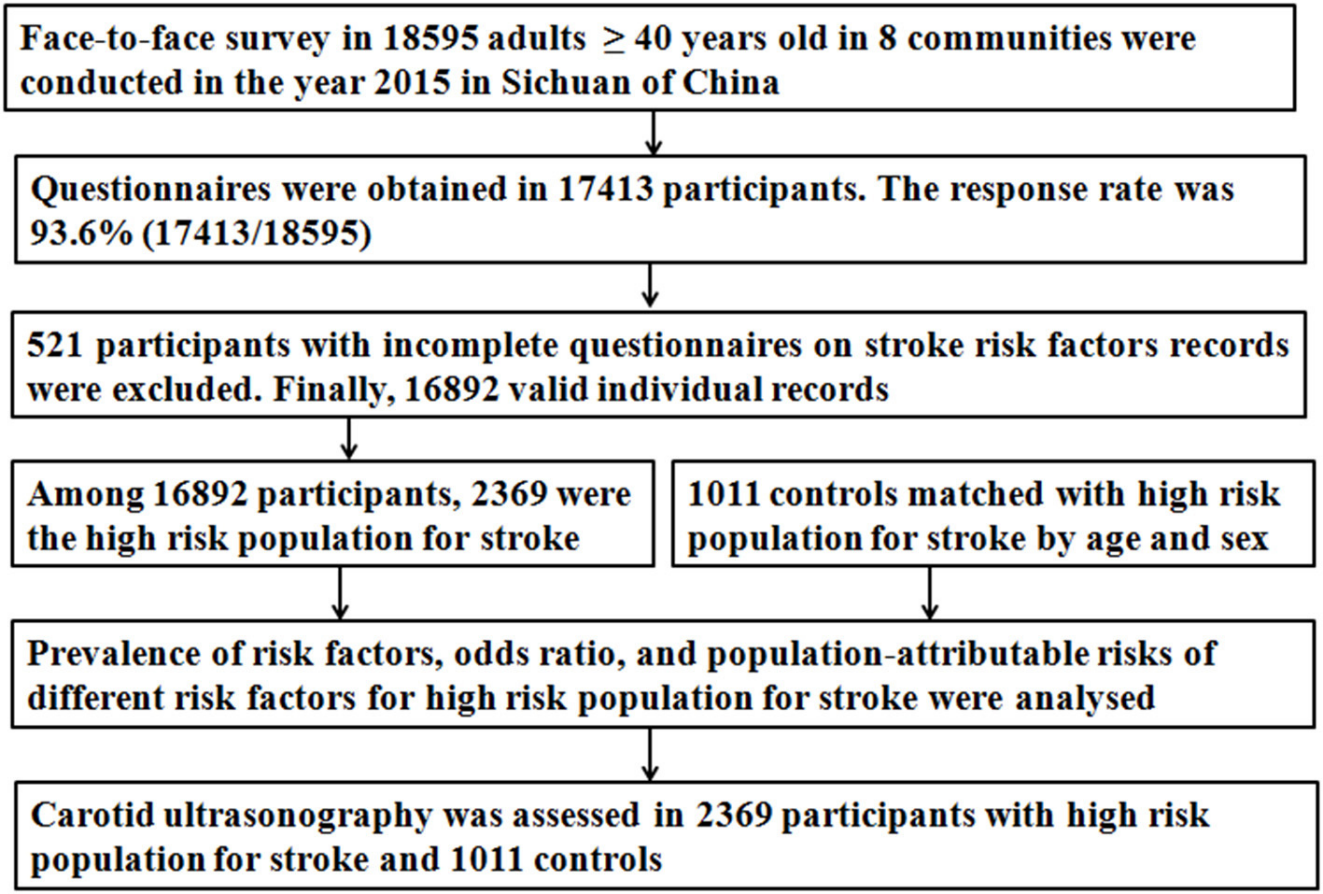
Data preparing and cleaning process in this survey.

The detailed data cleaning procedure were presented in Figure 1. Briefly, 18,595 participants participated in a face-to-face survey and questionnaires were obtained from 17,413 participants. The response rate was 93.6% (17,413/18,595). The 521 participants with incomplete questionnaires on stroke history or risk factors records (i.e., hypertension, diabetes, dyslipidemia, AF, overweight, smoking, physical inactivity, and family history of stroke) were excluded. Finally, 16,892 valid individual records were completed. Among 16,892 participants, 2,369 were classified as a high-risk population for stroke.

The interviewers were physicians or neurologists from community hospitals, who had at least 5 years of education in medicine. The quality of the measurements and data collection were maintained by implementing uniform training and standardized protocols. The staff involved in the survey were trained by the CNSSS program and passed the examination at the end of the train.

According to the CNSSS program, carotid characteristics were assessed by high-resolution B-mode ultrasound in the high-risk population for stroke in the second survey stage (21, 28). To determine the difference in carotid characteristics between high-risk and low-risk populations for stroke, 1,011 controls were selected among the low-risk population for stroke in the 8 communities using 2:1 matched case-control method as the age and sex matched, according to the criteria: (1) age ≥ 40 years; (2) age and sex were matched with high-risk stroke population; (3) carotid was assessed by high-resolution B-mode ultrasound.

### Carotid Ultrasonography

Bilateral common and internal carotid arteries, and bifurcations, were examined using a diagnostic ultrasound device (type 512, Acuson Sequoia Apparatus, 7.5-MHz probe, Berlin, Germany) in 2,125 high-risk populations for stroke (244 were unwilling to accept the examination) and 1,011 low-risk population for stroke, according to standard scanning and reading protocols (30, 31). Carotid characteristics, namely, intima-media thickness (IMT), plaque morphology, and degree of carotid stenosis were evaluated. The detailed procedures for evaluating plaques, types of plaques, degree of carotid stenosis, IMT, and intraobserver and interobserver coefficients were described in our previous study (30, 32). Carotid characteristics were graded independently by an ultrasound imaging doctor blinded to the clinical status of participants.

### Statistical Analysis

According to the CNSSS program, the survey should cover at least 1% of the local residents. According to the sixth national population census in 2010, there were 167,553 residents aged ≥ 40 years in the eight communities (33), 10% of the targeted population, therefore, the expected sample size was 16,755. Furthermore, the sample size (*N*) necessary for this survey was calculated based on a prevalence (*p*) of stroke of 2.37% among adults aged ≥ 40 years in China (34), with a 0.5% uncertainty level (*d*), using the formula *n* = tα^2^*pq*/*d*^2^ (*t* = 1.96, α = 95% for both sides; *q* = 1 – *p*). We calculated a required sample size of 16,765, considering a loss to follow-up rate of 10%, the planned sample size was 18,628 (16,765/0.90). Finally, 18,595 participants aged ≥ 40 years participated in this survey (Figure 1).

Descriptive analyses were conducted to determine the distribution of the demographic data and risk factors in the study population using SPSS 17.0 (SPSS Incorporation, Chicago, Illinois, USA). Categorical variables are presented as proportions and were compared using the chi-squared tests between different subgroups. The adjusted odds ratios (ORs) and 95% CIs of each risk factor for stroke prevalence rate were derived using unconditional multivariate logistic regression models, fully adjusting for all other potential confounders, namely, age, sex, education, smoking, urban/rural residency, physical inactivity, overweight, hypertension, dyslipidemia, diabetes, AF, and family history of stroke.

We calculated population-attributable risks (PARs) of the high-risk population for stroke from the model using the Bruzzi method for determining the confounder-adjusted PAR (35). The 95% CIs were evaluated for the PARs according to the previously described procedure (36). All the tests were two-sided and *P* < 0.05 was considered as statistically significant.

## Results

### Baseline Characteristics of Survey Population

In total of 16,892 participants, 2,369 (14.0%) were high-risk population for stroke. As shown in Table 1, the prevalence of high-risk population for stroke increased with age (*P* < 0.001), but decreased with educational level (*P* < 0.001). In addition, the prevalence of high-risk population for stroke was significantly higher in men than women and rural residents than urban residents (*P* < 0.001) (Table 1).

**Table 1:** Demographic characteristics of study population.

**Variables**	**Survey population, *n***	**Low-risk** **population for** **stroke, *n* (%)**	**High-risk** **population for** **stroke, *n* (%)**
Total	16,892	1,011	2,369 (14.0)
Sex			
Male	5,411	512 (9.5)	1,169 (21.6)^*^
Female	11,481	490 (4.3)	1,200 (10.5)
Age, y			
40–49	3,524	119 (3.4)	281 (7.9)^*^
50–59	5,106	266 (5.2)	583 (11.4)
60–69	5,803	376 (6.5)	897 (15.5)
70–79	2,183	204 (9.3)	498 (22.8)
≥80	276	46 (16.7)	110 (39.8)
Residence			
Urban	8,889	495 (5.6)	1,101 (12.4)^*^
Rural	8,003	516 (6.4)	1,268 (15.8)
Education			
Primary school or below	8,331	661 (7.9)	1,579 (18.9) ^*^
Junior middle school	3,312	197 (5.9)	462 (13.9)
Senior middle school	3,111	87 (2.8)	195 (6.3)
College or above	2,138	66 (3.1)	133 (6.2)
Overweight/obesity			
Yes	8,615	406 (4.7)	1,190 (13.8) **^#^**
No	8,277	605 (7.3)	1,179 (14.2) **^#^**
Smoking			
Yes	3,676	145 (3.9)	609 (16.6) ^#^^*^
No	13,216	866 (6.6)	1,760 (13.3) **^#^**
Physical inactivity			
Yes	8,226	451 (5.5)	1,447 (17.6) ^#^^*^
No	8,666	560 (6.5)	922 (10.6) **^#^**
Hypertension			
Yes	7,018	506 (7.2)	1,769 (25.2) ^#^^*^
No	9,874	505 (5.1)	600 (6.1) **^#^**
Diabetes			
Yes	2,754	153 (5.6)	674 (24.4) ^#^^*^
No	14,138	858 (6.1)	1,695 (11.9) **^#^**
Dyslipidemia			
Yes	3,235	132 (4.1)	681 (21.1) ^#^^*^
No	13,657	879 (6.4)	1,688 (12.4) **^#^**
TC (mM)	5.05 ± 1.54	5.08 ± 1.17	5.23 ± 1.33 **^#^**
LDL-C (mM)	2.56 ± 1.23	2.59 ± 1.14	2.98 ± 1.11 **^#^**
TG (mM)	1.65 ± 0.9	1.67 ± 0.6	1.82 ± 0.8 **^#^**
Atrial fibrillation			
Yes	232	6 (2.6)	58 (25.0) ^#^^*^
No	16,660	1,005 (6.0)	2,311 (13.9) **^#^**
Family history of stroke			
Yes	2,224	51 (2.3)	442 (19.9) ^#^^*^
No	14,668	960 (6.5)	1,927 (13.1) **^#^**

*TC, total cholesterol; LDL-C, low-density lipoprotein cholesterol; TG, triglycerides*.

# *P < 0.01, compared with low-risk population for stroke*;

* *P < 0.001, intragroup comparison in high risk population for stroke*.

Stratified by the eight risk factors for stroke, the prevalence of the high-risk population for stroke was the highest in the individuals with hypertension (25.2%), followed by those with AF (25.0%), diabetes (24.4%), dyslipidemia (21.1%), family history of stroke (19.9%), physical inactivity (17.6%), and smoking (16.6%) (*P* < 0.001, Table 1). The prevalence of high-risk population for stroke showed significant differences according to the eight stratified risk factors.

Among the low-risk population for stroke, we randomly selected 1,011 population (controls) by age and sex were matched with the high-risk population for stroke. The results showed that the prevalence of all eight risk factors and levels of TC, TG, and LDL-C were significantly higher in the high-risk population for stroke than the low-risk population for stroke (all *P* < 0.001, Table 1).

Furthermore, we conducted a subgroup analysis evaluating stroke risk factors by residential areas and sex. The prevalence of overweight/obesity, smoking, dyslipidemia, family history of stroke was higher, educational level, the proportion of antihypertensive treatment, hypoglycemic treatment, and statins were lower in rural residents than in urban residents (Table 2). The prevalence of overweight/obesity, smoking, physical inactivity, hypertension, dyslipidemia was higher, the proportion of antihypertensive treatment, hypoglycemic treatment, and statins was lower in men than women (Table 3).

**Table 2:** Stroke risk factors comparison between the rural and urban areas.

**Variables**	**Urban areas** **(*n* = 1,101)**	**Rural areas** **(*n* = 1,268)**	***P* value**
Male, *n* (%)	550 (50.0)	619 (48.8)	0.536
Age, y, *n* (%)			0.998
40–59	401 (36.4)	463 (36.5)	
≥60	700 (63.6)	805 (63.5)	
Education, *n* (%)			< 0.001
Junior middle school or below	881 (80.0)	1,160 (91.5)	
Senior middle school or above	220 (20.0)	108 (8.5)	
Overweight/obesity, *n* (%)	495 (45.0)	695 (54.8)	< 0.001
Smoking, *n* (%)	220 (20.0)	389 (30.7)	< 0.001
Physical inactivity, *n* (%)	720 (65.4)	727 (57.3)	< 0.001
Hypertension, *n* (%)	830 (75.4)	939 (74.1)	0.473
Antihypertensive drugs, *n* (%)	275(25.0)	255 (20.1)	0.004
Diabetes, *n* (%)	315 (28.6)	359 (28.3)	0.893
Hypoglycemic drugs, *n* (%)	110 (10.0)	88 (6.9)	0.008
Dyslipidemia, *n* (%)	266 (24.2)	415 (32.7)	< 0.001
Statins, *n* (%)	112 (10.2)	89 (7.0)	0.006
Atrial fibrillation, *n* (%)	27 (2.5)	31 (2.4)	0.999
Family history of stroke, *n* (%)	187 (17.0)	255 (20.1)	< 0.001

**Table 3:** Stroke risk factors comparison between men and women.

**Variables**	**Men** **(*n* = 1,169)**	**Women** **(*n* = 1,200)**	***P* value**
Age, y, *n* (%)			0.261
40–59	413 (35.3)	451 (37.6)	
≥60	756 (64.7)	749 (62.4)	
Rural areas	618 (52.9)	650 (54.2)	0.534
Education, *n* (%)			0.996
Junior middle school or below	1,007 (86.1)	1,034 (86.2)	
Senior middle school or above	162 (13.9)	166 (13.8)	
Overweight/obesity, *n* (%)	630 (53.9)	560 (46.7)	<0.001
Smoking, *n* (%)	378 (32.3)	231 (19.3)	<0.001
Physical inactivity, *n* (%)	760 (65.1)	687 (57.3)	<0.001
Hypertension, *n* (%)	935 (80.0)	834 (69.5)	<0.001
Antihypertensive drugs, *n* (%)	230 (19.7)	300 (25.0)	0.001
Diabetes, *n* (%)	340 (29.1)	334 (27.8)	0.472
Hypoglycemic drugs, *n* (%)	82 (7.0)	116 (9.7)	0.023
Dyslipidemia, *n* (%)	368 (31.5)	313 (26.1)	0.007
Statins, *n* (%)	84 (7.2)	117 (9.8)	0.026
Atrial fibrillation, *n* (%)	30 (2.6)	28 (2.3)	0.724
Family history of stroke, *n* (%)	222(19.0)	220 (18.3)	0.711

### Risk Factors for High-Risk Stroke Population

The multivariate logistic regression analysis was used to evaluate the risk factors for high-risk population for stroke. After full adjustments, all the eight risk factors and high level of TC, TG, and LDL-C were significantly associated with high-risk population for stroke (all *P* < 0.001, Table 4). The strongest risk factors for the high-risk population for stroke were hypertension (OR = 3.46, 95% CI = 1.82–6.13) and physical inactivity (OR = 2.45, 95% CI = 1.72–4.47), followed by dyslipidemia (OR = 2.27, 95% CI = 1.56–3.36), overweight/obesity (OR = 1.66, 95% CI = 1.35–2.42), diabetes (OR = 1.54, 95% CI = 1.33–2.28), family history (OR = 1.41, 95% CI = 1.26–1.95), smoking (OR = 1.35, 95% CI = 1.23–1.74), and AF (OR = 1.28, 95% CI = 1.14–1.63) (Table 4).

**Table 4:** Odds ratios and population-attributable risk factors for the high-risk stroke population by the multivariable regression models.

**Category**	**Reference groups**	**Control groups**	**Odds ratio (95% CI)**	***P* value**	**PAR (%) (95% CI)**
Sex	Female	Male	1.13 (0.97–1.52)	0.286	NA
Age, y	40–49	50–59	1.22 (1.07–1.73)	0.034	NA
		60–69	2.05 (1.58–3.92)	0.018	NA
		70–79	3.96 (2.17–5.38)	0.003	NA
		≥80	4.25 (2.28–6.27)	<0.001	NA
Residence	Rural	Urban	0.86 (0.76–1.13)	0.486	NA
Education	Primary school or below	Junior middle school	1.11 (0.92–1.36)	0.522	NA
		Senior middle school	1.03 (0.83–1.27)	0.433	NA
		College or above	0.92 (0.79–1.06)	0.359	NA
Overweight/obesity	No	Yes	1.66 (1.35–2.42)	0.006	9.4 (7.89–10.87)
Smoking	No	Yes	1.35 (1.23–1.74)	0.029	5.9 (4.36–6.97)
Physical inactivity	No	Yes	2.45 (1.72–4.47)	<0.001	14.2 (12.81–24.72)
Hypertension	No	Yes	3.46 (1.82–6.13)	<0.001	28.4 (25.63–35.92)
Diabetes	No	Yes	1.54 (1.33–2.28)	0.016	7.2 (6.13–8.05)
Dyslipidemia	No	Yes	2.27 (1.56–3.36)	<0.001	11.2 (9.23–14.62)
High TC	No	Yes	2.24 (1.52–3.17)	<0.001	10.5 (8.98–12.7)
High LDL-C	No	Yes	2.46 (1.65–4.54)	<0.001	13.6 (11.3–17.78)
High TG	No	Yes	1.34 (1.08–1.53)	0.043	4.1 (3.12–4.64)
Atrial fibrillation	No	Yes	1.28 (1.14–1.63)	0.031	3.5 (3.16–4.88)
Family history	No	Yes	1.41 (1.26–1.95)	0.022	6.8 (6.21–7.99)

*CI, confidence interval; NA, not applicable; PAR, population-attributable risk; TC, total cholesterol; LDL-C, low-density lipoprotein cholesterol; TG, triglycerides*.

After full adjustments, hypertension (28.4%), physical inactivity (14.2%), and dyslipidemia (11.2%) were three risk factors with the largest contributions to the PAR of the high-risk population for stroke. Diabetes, overweight/ obesity, family history, smoking, and AF accounted for < 10% of the total PAR of the high-risk population for stroke (Table 4).

### Comparisons of Carotid Characteristics Between High- and Low-Risk Populations for Stroke

As shown in Table 5, compared with the low-risk population for stroke, the proportion of IMT > 1 mm in the common carotid artery, carotid stenosis > 50%, and carotid plaques including echolucent plaque or echogenic plaque were significantly higher in the high-risk population for stroke (*P* < 0.001, *P* = 0.021, and *P* < 0.001, respectively), these indicated that carotid atherosclerosis was more serious in a high-risk population for stroke compared with low-risk population for stroke.

**Table 5:** Comparison of carotid atherosclerosis between the high-risk stroke population and low-risk stroke population.

**Variables**	**Low-risk stroke population** **(*n* = 1,011)**	**High-risk stroke population** **(*n* = 2,125)**	***P* value**
IMT > 1 mm in common carotid artery, *n* (%)	91 (9.0)	419 (19.7)	<0.001
Carotid plaques, *n* (%)			<0.001
Nonplaque	841 (83.1)	1,383 (65.1)	
Echogenic plaque	102 (10.1)	399 (18.8)	
Echolucent plaque	68 (6.7)	343 (16.1)	
Carotid stenosis > 50%, *n* (%)	8 (0.8)	40 (1.9)	0.021

*IMT, intima-media thickness*.

## Discussion

Based on the CNSSS program in China, we conducted this survey, and provided more recent and up-to-date data on the prevalence of high-risk populations for stroke and the contribution of risk factors for the high-risk population in Sichuan. The results showed that the prevalence of high-risk population for stroke was very high, all eight risk factors for stroke were significantly associated with the high-risk population for stroke, the largest contributor was hypertension (PAR 28.4%), followed by physical inactivity, dyslipidemia, overweight/ obesity, diabetes, family history, smoking, and AF.

Hypertension is the most important risk factor and contributor to stroke (6, 12). The prevalence of hypertension substantially increased from 1979 to 2014 (national average prevalence of 28% between 2013 and 2014) in China, and there has been a geographical gradient in the prevalence of hypertension, which is highest in northeast China (13, 14). Despite the proportions of awareness and the treatment and control of hypertension are improving in recent years, the proportion of people whose hypertension is controlled is <20% in China, which is significantly lower than in the USA or the UK (14, 15). In this survey, we found that the prevalence of hypertension was 41.5% (7,018/16,892) in Sichuan, it was higher than the national average prevalence of 28% (13), and hypertension was the largest contributor for the high-risk population for stroke. However, only 29.7% (2,082/7,018) of patients with hypertension were receiving antihypertensive treatment in the survey population. The high prevalence of hypertension and low proportion of antihypertensive treatment may be important causes of the high prevalence of the high-risk population for stroke in Sichuan. These data emphasize the crucial importance of improving blood pressure control.

Physical inactivity was ranked as the second important risk factor among the eight risk factors, with PAR of 14.2%, followed by dyslipidemia, overweight/ obesity, diabetes, family history of stroke, smoking, and AF in this survey. These indicate that China has experienced rapid sociodemographic changes and health transitions, the prevalence of major risk factors for stroke has changed due to comprehensive changes in lifestyle and dietary habits that have occurred in recent years.

Physical inactivity has been proved to be associated with an increased risk for stroke (37). The protective effect of physical activity may play an important role in reducing blood pressure and blood lipid levels and controlling other risk factors for stroke, such as diabetes and overweight/obesity (38, 39). However, the population in physical inactivity increased by 25% from 1991 to 2011, consequently increasing the risk of being overweight/obesity and the related metabolic abnormalities in the Chinese population (40). Smoking was another behavioral risk factor for stroke, which has been widely acknowledged nowadays. In contrast to previous studies (41), smoking only accounted for 5.9% of the PAR for the high-risk population for stroke in this study. This may be due to the significant decrease in the prevalence of smoking from 30.4% in 1980 to 24.2% in 2012 (42). Based on the findings, a healthy lifestyle is very important in reducing the prevalence of the high-risk population for stroke.

In this study, it was shown that metabolic factors (i.e., dyslipidemia, diabetes, and overweight/obesity) were also associated with the high-risk population for stroke. The dietary habit of the population may affect the risk factors. The traditional Chinese diet, characterized by high intakes of refined cereal products, salted vegetables, sodium, fat and red meat and low intakes of fruit and vegetables, is associated with an elevated risk factors for stroke. Recent studies showed that many diet-related risk factors, namely, hypertension, overweight/obesity, dyslipidemia, and diabetes increased in the past several decades in China. Prevalence of dyslipidemia increased from 8% in 1985 to 11.2% in 2014 in China (41, 43). Increased energy intake of fat may increase obesity and dyslipidemia, consequently increasing the risk of related metabolic abnormalities (44). High sodium intake is associated with an increased risk of both hypertension and stroke (13). Changing lifestyles, namely, increased consumption of red meat, low consumption of vegetables and fruit, and physical inactivity, have resulted in the rapidly rising rates of diabetes in China (45). Although personal daily consumption of fruit and vegetables among the Chinese population has gradually increased since the 1980s, consumption is still below nationally recommended amounts. According to our results, the proportions of hypoglycemic treatment and statins were very low in the population with diabetes and dyslipidemia. Thus, clinical control of dyslipidemia and diabetes, healthy dietary habits are necessary for preventing high-risk populations for stroke (46).

Previous studies demonstrated that the prevalence of stroke was significantly higher in men than in women, in rural population than in the urban population in China (6, 12). This study showed that the prevalence of high-risk population for stroke was significantly higher in men than in women, in rural residents than in urban residents. This may result from the high prevalence of risk factors for stroke and a low proportion of antihypertensive treatment, hypoglycemic treatment, and statins in men than in women, in rural residents than in urban residents. These alarming trends indicate the need for better interventions for these vulnerable populations.

Numerous studies have shown that carotid atherosclerosis is a very important etiology and a risk factor for stroke (30–32, 47). In this study, we found that the prevalence of IMT thickening (IMT > 1 mm), vulnerable plaque, and ≥ 50% carotid stenosis was higher in the high-risk population for stroke than in the low-risk population for stroke. The results suggest that it is important to control risk factors for stroke and reduce the high-risk stroke population for preventing carotid atherosclerosis occurrence and development.

This was a multicenter, cross-sectional, and community-based high-risk stroke population survey involving a large representative sample of Sichuan in southwestern China. We identified a high prevalence of high-risk population for stroke and contribution of risk factors for high-risk population, and high prevalence of IMT thickening, vulnerable plaque, and ≥ 50% carotid stenosis among adults aged ≥ 40 years in southwestern China. This survey is interesting because it underlines the importance of accompanying economic improvement with educational interventions both for the population and for doctors to avoid a simultaneous increase in known risk factors for stroke. These results are also very important for the primary prevention of stroke.

Although this was the most recent survey of the high-risk population for stroke and associated risk factors, this cross-sectional survey involved a large representative sample of Sichuan in southwestern China. This study has several limitations. First, this survey only sampled residents for age ≥ 40 years; therefore, our current results cannot be generalized to all the population groups in southwestern China. Second, this study was a cross-sectional study, and there may have been a recall bias because of the self-reported questionnaire. Moreover, although of large sample size, the nature of the cross-sectional study design, we did not eliminate biases of the calculation of PAR for risk factors. Thus, follow-up of the participants is necessary for the future. Third, some other risk factors (such as alcohol intake, air pollutants, and dietary patterns) are shown to contribute to stroke risk. However, we were unable to involve them in the analyses due to the lack of this information in this survey. Finally, this study was the absence of prospective follow-up of participants and longitudinal outcomes data in this stage, these may make the calculation of PARs of limited value. Thus, we will follow up with the participants and analyze the prevalence of stroke to fill the important gap in our future studies.

## Conclusion

In this study, we have identified a high prevalence of high-risk population for stroke, and contribution of risk factors for high-risk population among adults aged ≥ 40 years in southwestern China. All the eight risk factors for stroke were significantly associated with the high-risk stroke population; the largest contributor was hypertension, followed by physical inactivity, dyslipidemia, overweight/obesity, diabetes, family history, smoking, and AF. Carotid atherosclerosis was more serious in the high-risk stroke population. Thus, individual-level and population-level interventions for these leading risk factors are very important for the primary prevention of stroke.

## Data Availability Statement

The original contributions presented in the study are included in the article/supplementary material, further inquiries can be directed to the corresponding authors.

## Ethics Statement

The studies involving human participants were reviewed and approved by the Ethics Committee of People's Hospital of Deyang City (IRB number: 2015-024). The patients/participants provided their written informed consent to participate in this study.

## Author Contributions

XY, HC, and SB: designed the study and acquired funding. YW, MY, WW, and XC: performed this survey. SB, XY, and WW: analyzed the results and drafted the figure. XC, SB, and HL: drafted the tables and manuscript. XY, SB, MY, and HL: supervised the project. All authors bear responsibility for the integrity and accuracy of the data in the study. All authors contributed to the article and approved the submitted version.

## Funding

This study was supported in part by grants from the Scientific Research Foundation of Sichuan Provincial Health Department (Grant No. 16ZD046) and the Sichuan Science and Technology Agency Research Foundation (Grant No. 2018JY0164).

## Conflict of Interest

The authors declare that the research was conducted in the absence of any commercial or financial relationships that could be construed as a potential conflictof interest.

## Publisher's Note

All claims expressed in this article are solely those of the authors and do not necessarily represent those of their affiliated organizations, or those of the publisher, the editors and the reviewers. Any product that may be evaluated in this article, or claim that may be made by its manufacturer, is not guaranteed or endorsed by the publisher.
